# The six-minute step test can predict COPD exacerbations: a 36-month follow-up study

**DOI:** 10.1038/s41598-024-54338-9

**Published:** 2024-02-13

**Authors:** Aldair Darlan Santos-de-Araújo, Cássia da Luz Goulart, Renan Shida Marinho, Izadora Moraes Dourado, Renata Gonçalves Mendes, Meliza Goi Roscani, Daniela Bassi-Dibai, Shane A. Phillips, Ross Arena, Audrey Borghi-Silva

**Affiliations:** 1 Cardiopulmonary Physiotherapy Laboratory, Universidade Federal de São Carlos, São Carlos, SP 13565-905 Brazil; 2 Department of Medicine, Universidade Federal de São Carlos (UFSCar), Sao Carlos, SP Brazil; 3 Postgraduate Program in Management in Health Programs and Services, Universidade CEUMA, São Luís, MA Brazil; 4 Department of Physical Therapy, College of Applied Health Sciences, University of Illinois Chicago, Chicago, IL USA

**Keywords:** Chronic obstructive pulmonary disease, Functional capacity, Six-minute step test, Exacerbation, Diseases, Respiratory tract diseases

## Abstract

The six-minute step test (6MST) has been shown to be effective in assessing exercise capacity in individuals with COPD regardless of severity and, despite its easy execution, accessibility and validity, information on the prognostic power of this test remains uncertain. The aim of this study is to investigate whether the 6MST can predict the occurrence of exacerbations in patients with COPD. This is a prospective cohort study with a 36-month follow-up in patients with COPD. All patients completed a clinical assessment, followed by pulmonary function testing and a 6MST. The 6MST was performed on a 20 cm high step; heart rate, blood pressure, oxygen saturation, BORG dyspnea and fatigue were collected. Sixty-four patients were included in the study, the majority being elderly men. Performance on the 6MST demonstrated lower performance compared to normative values proposed in the literature, indicating a reduced functional capacity. Kaplan Meier analysis revealed that ≤ 59 steps climbed during the 6MST was a strong predictor of COPD exacerbation over a 36-month follow-up. We have identified a minimal threshold number of steps (≤ 59) obtained through the 6MST may be able predict the risk of exacerbations in patients with COPD.

## Introduction

Impaired respiratory function in patients with chronic obstructive pulmonary disease (COPD) commonly leads to exertional dyspnea, hypoxemia and hypercapnia, which contributes to the diminished ability to perform activities of daily living and disability seen in this population^1,2^. The extrapulmonary pathological multisystem characteristics of COPD also leads to skeletal muscle weakness^3^, sarcopenia^4^, further contribting to the reduction in exercise capacity^5^.

Incontestably, the scientific literature has consistently emphasized that individuals with COPD experience exercise intolerance and functional impairment, reflected in impairments in activities of daily living^5^. Not surprisingly, the assessment of exercise capacity in this population has been the subject of numerous investigations with an overarching goal of determining clinical utility (i.e., how does exercise capacity assessment contribute to therapeutic decision-making)^6–8^. Cardiopulmonary exercise test (CPET) is the gold standard assessment of exercise capacity. However, cost and expertise constraints of CPET limit availability in certain settings. As such, low-cost and readily available approaches to exercise capacity assessment are needed^9,10^.

The six-minute walk test (6MWT)^10^ has a longstanding history of being a valid and reproducible tool in the clinical and research settings in patients with COPD. Moreover, other tools, such as the six-minute step test (6MST), were introduced as an alternative option when space needed to perform the 6MWT is limited^10,11^. By adding the antigravity displacements inherent to 6MST, the act of going up and down a step causes large muscle groups, especially in the lower limbs, to be subjected to more intense physiological responses and greater oxygen consumption in a significantly shorter time when compared to the 6MWT^12^. The 6MST has been shown to be valid, reproducible and strongly correlated with the 6MWT and CPET^11^. The 6MST has also been shown to be effective in assessing exercise capacity in individuals with COPD regardless of severity^11^. At this point, the prognostic utility of the 6MST remains largely unexplored. In this context, the goal of the current investigation was to assess the ability of the 6MST to predict COPD exacerbations over a 36-month follow-up under the hypothesis that a minimum number of steps derived from the functional performance of the test can be a significant predictor of exacerbation.

## Metodology

### Study design

This is a prospective cohort study with a three-year follow-up (36 months) conducted according to the STrengthening the Reporting of OBservational studies in Epidemiology (STROBE). The current study was carried out at the Federal University of São Carlos (São Carlos, SP, Brazil), approved by the Research Ethics Committee of the institution (protocol number: 5.188.654) and conducted according to Declaration of Helsinki. All participants were informed of the purpose of the study and informed consent was obtained.

### Participants

Eligible patients were recruited from the Pneumology Outpatient Clinics of the Center for Medical Specialties (CEME) and the University Hospital of São Carlos (HU-UFSCar) in addition to those recruited from the Health School Unit of the Federal University of São Carlos (UFSCar). Patients of both sexes, aged > 40 years, with a clinically stable (i.e., without exacerbation for at least 3 months) diagnosis of COPD confirmed by previous pulmonary function test results, and were prescribed an optimized medication regimen were included.

Exclusion criteria consisted of: (1) > 80 years of age; (2) current history of alcoholism; (3) thyroid disorders; (4) reduced left ventricular ejection (< 50% on echocardiogram); (5) history of cardiac events (myocardial infarction and cardiac surgery) in the last 6 months; (6) diagnosis of heart failure; (7) implantable pacemaker; (8) unstable angina; (9) history of cardiac arrhythmias; (10) diagnosis of lung cancer or other types of cancer; (11) uncontrolled hypertension; (12) uncontrolled diabetes mellitus; (13) renal failure; (14) the inability to perform lung function testing; and (15) refusal to participate in the study.

### Body composition analysis

Body composition analysis was performed using an InBody 720 device electrical bioimpedance scale (Biospace co, Seoul, Korea). Subjects were instructed to urinate before the test, not to drink alcohol and not to perform strenuous exercises the day before the test. During the evaluation, subjects remained in orthostasis, wearing light clothes, barefoot, shoulders slightly abducted and elbows flexed at approximately 15°, following the manufacturer's recommendations. Measures related to skeletal muscle mass (kg), body fat mass (kg and %), body weight (kg) and body mass index (BMI) (kg/m^2^) were collected.

### Six-minute step test—6MST

Prior to the test, subjects remained seated for two minutes and then were instructed to remain standing while vital signs were collected (resting heart rate [HR], peripheral oxygen saturation [SpO_2_] and systemic blood pressure) in addition to the perception of exertion for dyspnea and lower limb fatigue using the BORG 10 scale throughout the test. The individuals were instructed to go up and down a single step at a height of 20 cm (cm) at their self-select pace and, if necessary, rest as needed. The individuals could alternate the lower limbs but upper limbs had no support and remained stationary alongside the body^11^. At each minute of the test, verbal commands of encouragement were given, and the subjects were informed every minute about the remaining time until the test was finished. The entire number of steps were for the 6-min test were recorded. Heart rate, systemic blood pressure, SpO_2_, dyspnea sensation and lower limb fatigue scores were obtained immediately at the end of the test, in the first, third and last minute of recovery. Due to its submaximal nature, some test interruption criteria were adopted so that the integrity of the patient's health was guaranteed, including: (1) reaching 85% of the maximum HR; (2) arterial oxygen saturation ≤ 87%; (3) systolic blood pressure (SBP) greater than 170 mmHg and DBP greater than 110 mmHg; (4) BORG scores greater than 7 for dyspnea and lower limb fatigue; (5) anginal pain > 2; and (6) dizziness, vertigo and nausea. If there was a need for interruption and test time was available, subjects were encouraged to resume test performance when clinical signs stabilized. A performance ≤ 78 steps was used as an indication of decreased functional capacity^11^. To predict functional performance for the Brazilian population, the equations proposed by Arcuri et al.^13^ and Albuquerque et al.^14^ were considered.

### Spirometry

The spirometry was evaluated using a calibrated spirometry (Masterscreen Body, Mijnhardt/Jäger, Würzburg, German) by a previously trained researcher, which used conventional techniques and followed the technical recommendations of acceptability and reproducibility from the American Thoracic and European Respiratory Societies (ATS/ERS)^15^. At least three slow, forced, acceptable and reproducible maneuvers were performed and repeated 20 min after the inhalation of Albuterol Sulfate (400 µg). The GOLD criteria^16^ [post-bronchodilator forced expiratory volume in the 1 s (FEV_1_)/forced vital capacity (FVC) ratio < 0.70] was used to confirm a COPD diagnosis. The volunteers were classified into their airflow limitation severity stage based on their percentage of predicted FEV_1_ using GOLD guidelines: I (mild): FEV_1_ ≥ 80% predicted; II (moderate): 50% ≤ FEV_1_ < 80% predicted; III (severe): 30% ≤ FEV_1_ < 50% predicted; and IV (very severe): FEV_1_ < 30% predicted^16^. The results were contrasted with those predicted by Pereira et al^17^.

### Modified medical research council (mMRC) dyspnea scale

This tool, consists of a 5-item questionnaire in which patients categorize their degree of disability, reflecting how dyspnea affects their mobility^18^. Patients reported their subjective degree of dyspnea by choosing a value between 0 and 4. Lower MMRC scores are associated with less impairment in activities of daily living related to dyspnea.

### Duke activity status index questionnaire—DASI

It is a questionnaire composed of 12 items that assess daily activities such as personal care, walking, housework, sexual activity, and recreational activities, estimating their respective metabolic expenditures. Simple, fast, easy to apply and validated for the Brazilian population^19^, the DASI questionnaire is used to calculate an estimated peak oxygen consumption (VO_2_). Each item has a score proportionally based on the metabolic equivalent (MET) for each activity. The final score ranges between 0 and 58.2 mL·kg^−1^·min^−1^, estimated using the following multiple linear regression equation^20^: VO_2_ = 0.43 × DASI + 9.6.

### Participants follow-up

To quantify and follow exacerbation events in the patients included in the study, phone calls were made periodically every six months to the patient after the date of the patient's initial evaluation in the laboratory.

### History of exacerbation

COPD exacerbation, according to the Global Initiative for Chronic Obstructive Lung Disease (GOLD), was considered when the patient experienced an acute episode characterized by worsening respiratory signs and symptoms, in addition to normal daily variations, such as a significant increase in resting dyspnea, persistent cough, production of purulent sputum and increased sputum volume, sufficient to require additional therapeutic management, such as change in regular medication or need for hospitalization^21^. Information on exacerbations was collected through telephone contact with patients during follow-up assessments as well as through hospital records.

#### Statistical analysis

Data are presented as mean and standard deviation or absolute values and percentages of occurrence when appropriate. Receiver operating characteristic (ROC) curve analysis: Cut-off points discriminated the precision of the number of steps in determining exacerbation risk. The 95% confidence interval (CI) was used to determine the predictive ability of the clinical variables, with the lower limit being greater than 0.50. Subsequently, the cut -off points of the variable that obtained significant area under the ROC curve were identified, with the respective values of sensitivity and specificity. ROC curve analyses selected the optimal threshold values (the Youden's J index = Sensitivity + Specificity − 1) for the for the number of steps achieved.

The Shapiro–Wilk test was used to verify the normality of the data. For the analysis between the groups (> 59 steps and ≤ 59 steps), Student's t-test was used when the assumptions of normality were met. The Mann–Whitney test was used when this assumption was violated. χ^2^ test was used to compare categorical variables values (gender, comorbidities, GOLD classification, medications and mMRC) among groups.

Kaplan–Meier analysis: all hospitalization that occurred during the 36-months follow-up were examined. Hospitalization curves were analyzed according to the Kaplan–Meier method to explore the impact of ≤ 59 steps. Differences between curves were evaluated using the Breslow and Tarone-Ware.

Cox proportional regression models [adjusted for steps by 6MST, gender, total body mass (kg) and body fat mass (kg)] were performed, with associations expressed as risk ratios (HRs) and 95% CIs.

All analyzes were performed using Statistical Package for the Social Sciences (SPSS) (IBM—SPSS, version 20.0 for Windows, Armonk, NY). The probability of type 1 error occurrence was established at 5% for all tests (*p* < 0.05).

## Results

Table 1 describes the general characteristics of the patients included in this study. Initially, 92 patients were recruited and evaluated. However, 9 could not perform the 6MST due to orthopedic limitations; 3 presented Spo2 < 87% during the test; 4 presented Heart Rate above 85% of the expected with lentified recovery; 5 refused to perform; and 7 did not complete the necessary evaluations over the follow-up. Finally, 64 patients were included in the study. The majority of the 64 patients were elderly men. In general, the individuals were hypertensive (49%), sedentary (45%) and ex-smokers (73%). There was a tendency towards reduced functional capacity according to the final score of the DASI questionnaire and a feeling of dyspnea grades 1 (44%) and 2 (23%) when evaluated using the mMRC scale. A wide variation of airflow obstruction was visualized in the entire sample when the spirometry results were interpreted. However, in short, the individuals present with moderate to severe airflow limitation, GOLD II (48.0%) and GOLD III (33.0%). Short-acting β-agonists (SABA) (63.0%), long-acting β-agonists (LABA) (35.0%), long-acting muscarinic antagonists (LAMA) (19.0%) and bronchodilators (38.0%) make up the list of most prevalent drugs in the sample.

**Table 1:** General characteristics of the study sample (n = 64).

Variables	COPD (n = 64)	> 59 steps (n = 39)	≤ 59steps (n = 25)	*p* value
Age (years)	66.00 ± 7.00	65.00 ± 6.00	66.00 ± 8.00	0.73
Gender				
Male, n (%)	40 (62.50)	25 (64.10)	15 (60.00)	0.74
Female, n (%)	24 (37.50)	14 (35.90)	10 (40.00)	
Total body mass (kg)	70.70 ± 20.10	67.10 ± 15.60	76.80 ± 24.80	0.05*
Height (m)	1.62 ± 0.09	1.63 ± 0.08	1.60 ± 0.10	0.23
BMI (kg/m^2^)	26.60 ± 6.77	25.00 ± 4.85	30.00 ± 8.52	< 0.01*
Body fat mass (kg)	22.36 ± 13.17	17.74 ± 8.56	31.34 ± 15.98	< 0.01*
Body fat (%)	31.38 ± 9.65	27.64 ± 7.66	38.88 ± 8.95	< 0.01*
Skeletal Muscle Mass (kg)	27.25 ± 9.67	27.25 ± 8.49	27.26 ± 11.91	0.99
Exacerbated Patient, n(%)	21 (32.81)	8 (20.51)	13 (52.00)	< 0.01*
Risk factors, n (%)				
Asma	12 (18.75)	6 (15.38)	6 (24.00)	0.39
Hypertension	31 (48.43)	16 (41.02)	15 (60.00)	0.10
Obesity	18 (28.12)	6 (15.38)	12 (48.00)	
Dyslipidemia	13 (20.31)	7 (17.94)	6 (24.00)	0.55
Type 2 Diabetes	4 (6.25)	2 (5.12)	2 (8.00)	0.61
Obstructive sleep Apnea syndrome	13 (20.31)	5 (12.82)	8 (32.00)	0.06
Current smokers	17 (26.56)	9 (23.07)	8 (32.00)	0.43
Ex-smokers	47 (73.43)	30 (76.92)	17 (68.00)	0.83
Tuberculosis sequelae	1 (1.56)	1 (2.56)	0 (0.00)	0.42
Sedentarism	29 (45.31)	14 (35.89)	15 (60.00)	0.06
Duke	32.40 (11.80)	35.00 (13.00)	28.00 (9.00)	0.03*
Duke (VO_2_)	23.50 (5.10)	24.50 (5.50)	21.60 (3.90)	0.04*
mMRC, n (%)				
0	9 (14.06)	9 (23.07)	0 (0.00)	< 0.01*
1	28 (43.75)	16 (41.02)	12 (48.00)	
2	15 (23.43)	11 (28.20)	4 (16.00)	
3	5 (7.81)	3 (7.69)	2 (8.00)	
4	7 (10.93)	0 (0.00)	7 (28.00)	
Pulmonary function				
FEV1 (L)	1.44 ± 0.65	1.59 ± 0.74	1.20 ± 0.38	0.04*
FEV1 (%)	59.28 ± 19.89	63.59 ± 20.20	52.56 ± 17.77	0.03*
FVC (L)	2.60 ± 0.87	2.76 ± 0.99	2.34 ± 0.58	0.16
FVC (%)	85.93 ± 20.24	89.11 ± 22.16	80.98 ± 16.00	0.09
FEV1/FVC	0.54 ± 0.11	0.56 ± 0.11	0.51 ± 0.10	0.11
Echocardiogram				
LVEF, %	68.00 ± 7.00	67.00 ± 6.00	68.00 ± 7.00	0.89
GOLD				
I (mild)	11 (17.19)	8 (20.51)	3 (12.00)	0.02*
II (moderate)	31 (48.44)	23 (58.97)	8 (32.00)	
III (severe)	21 (32.81)	8 (20.51)	13 (52.00)	
IV (very severe)	1 (1.56)	0 (0.00)	1 (4.00)	
Medications, n (%)				
SABA	40 (62.50)	23 (58.97)	17 (68.00)	0.41
LABA	22 (3.37)	11 (28.20)	11 (44.00)	0.17
LAMA	12 (18.75)	5 (12.82)	7 (28.00)	0.12
Bronchodilator	24 (37.50)	13 (33.33)	11 (44.00)	0.36
Antihypertensive	9 (14.06)	6 (15.38)	3 (12.00)	0.72
Diuretic	2 (3.12)	0 (0.00)	2 (8.00)	0.08
Calcium channel blockers	2 (3.12)	1 (2.56)	1 (4.00)	0.74

Values are mean ± SD or absolute values (%). COPD: Chronic Obstructive Pulmonary Disease; kg: kilos; m: meter; BMI: body mass index; VO2: oxygen uptake; mMRC: Modified Medical Research Council; FEV_1_: forced expiratory volume in 1 s; L: liters; FVC: forced vital capacity; GOLD: Global Initiative for Chronic Obstructive Lung Disease; SABA: short-acting β-agonist; LABA: long-acting β-agonist; LAMA: long-acting muscarinic antagonists. *p < 0.05 Statistical significance for Student’s t-test, Mann–Whitney test or χ^2^ test.

Table 2 presents the results of the patients' performance in the 6MST, demonstrating a lower performance than the cutoff point proposed in the literature (< 78 steps)^11^, as well as a lower performance than the predicted values for the healthy adults^13,14^ and consequent reduction in functional capacity.

**Table 2:** Functional capacity through 6MST (n = 64).

Variables	COPD (n = 64)	> 59 steps (n = 39)	≤ 59 steps (n = 25)	*p* value
Six-minute step test – 6MST				
Steps	74.0 ± 29.0	93.0 ± 19.0	45.0 ± 13.0	< 0.01*
% Predict by Arcuri et al. (2016)	59.0 ± 24.0	74.0 ± 17.0	36.0 ± 12.0	< 0.01*
% Predict by Albuquerque et al. (2022)	54.0 ± 21.0	67.0 ± 16.0	34.0 ± 11.0	< 0.01*
HR (bpm) rest	81.0 ± 14.0	76.0 ± 12.0	88.0 ± 14.0	< 0.01*
HR (bpm) peak	114.0 ± 18.0	114.0 ± 16.0	113.0 ± 22.0	0.12
HR (bpm) rec 1’	97.0 ± 17.0	95.0 ± 15.0	99.0 ± 19.0	0.90
SBP (mmHg) rest	122.0 ± 12.0	120.0 ± 10.0	125.0 ± 12.0	0.04*
SBP (mmHg) peak	155.0 ± 20.0	156.0 ± 22.0	153.0 ± 17.0	0.52
DBP (mmHg) rest	80.0 ± 9.0	79.0 ± 8.0	82.0 ± 10.0	0.16
DBP (mmHg) peak	91.0 ± 12.0	90.0 ± 14.0	92.0 ± 10.0	0.63
SpO_2_ (%) rest	95.0 ± 2.0	95.0 ± 2.0	93.0 ± 2.0	< 0.01*
SpO_2_ (%) peak	92.0 ± 4.0	93.0 ± 3.0	90.0 ± 4.0	< 0.01*
Δ SpO_2_ (%)	− 2.0 ± 3.0	− 2.0 ± 2.0	− 4.0 ± 3.0	< 0.01*
BORG Dyspnea rest	0.3 ± 1.0	0.3 ± 0.5	0.5.0 ± 1.0	0.08
BORG Dyspnea peak	4.0 ± 2.0	4.0 ± 3.0	5.0 ± 2.0	0.89
Δ BORG Dyspnea	4.0 ± 2.0	4.0 ± 3.0	4.0 ± 2.0	0.76
BORG fatigue lower limbs rest	0.3 ± 1.0	0.3 ± 1.0	0.5 ± 1.0	0.67
BORG fatigue lower limbs peak	5.0 ± 3.0	4.0 ± 2.0	5.0 ± 3.0	0.64
Δ BORG fatigue lower limbs	4.0 ± 3.0	4.0 ± 2.0	3.0 ± 2.0	0.53

Values are mean ± Standard Deviation. 6MST: six-minute step test; COPD: chronic obstructive pulmonary disease; HR: heart rate; SBP: systolic blood pressure; DBP: diastolic blood pressure; bpm: beats per minute; SpO2: peripheral oxygen saturation; mmHg: millimeters of mercury. *p < 0.05 Statistical significance for Student’s t-test or Mann–Whitney test.

When dividing subjects into > 59 steps (n = 39) and ≤ 59 steps (n = 25), homogeneity was observed between the groups, with no significant differences in terms of age, gender, risk factors and medications used. However, it can be observed that the group of patients who performed worse, that is, performed ≤ 59 steps, had significantly higher body mass (kg), fat mass (kg) and BMI. Furthermore, the group with poorer 6MST performance (< 59 steps) presented with a lower DASI score poorer performance on the mMRC, worse lung function, especially in absolute and percentage FEV1, and greater disease severity when considering the GOLD classification criteria. According to Table 2, The group of patients ≤ 59 steps presented significantly lower functional capacity (*p* = 0.04) when compared to the group of patients > 59 steps. When considering the predictive equations predicted by Arcuri et al.^13^ and Albuquerque et al.^14^, none of the groups managed to reach the predicted values, with a significant reduction, especially in the group with the worst performance (≤ 59 steps). Regarding hemodynamic responses, individuals in the lower 6MST performance group had considerably higher heart rate and systolic blood pressure at rest as well as a lower frequency of oxygen desaturation (SpO_2_) at rest and peak of the test.

**Figure 1:**
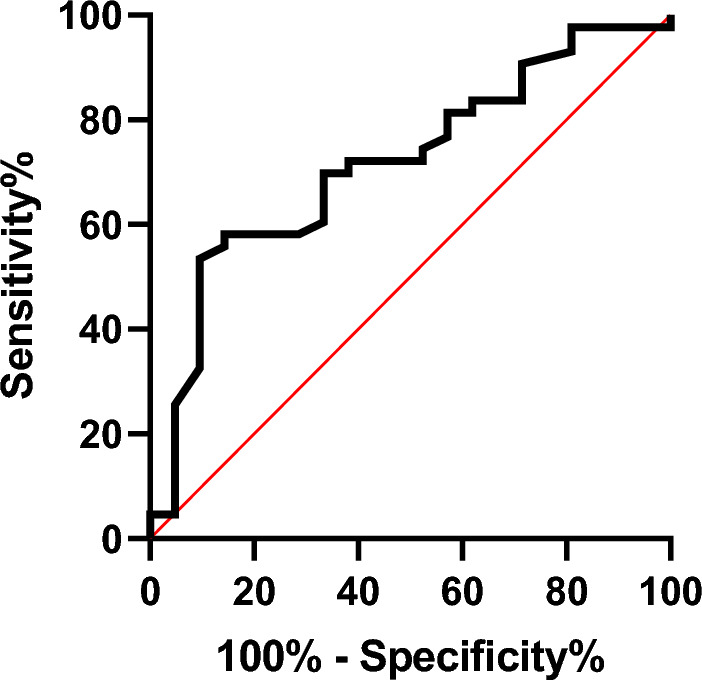
Receiver operating curves in patients with COPD. The area under the curve (AUC) of six-minute step test.

**Figure 2:**
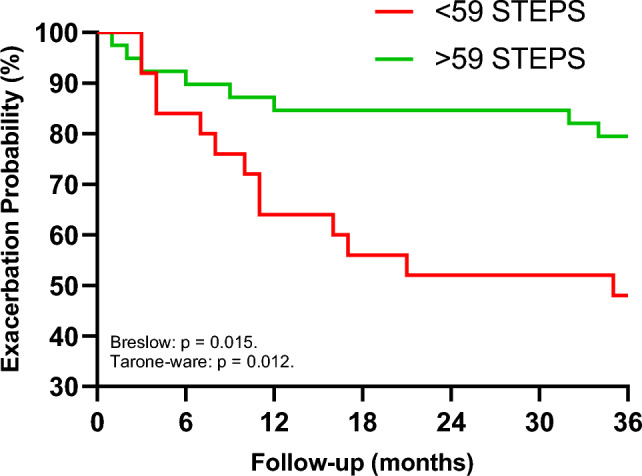
Kaplan–Meier curve for exacerbation according with functional capacity by cutoff point over a period of 36 months.

According to ROC curve analysis (Table 3 and Fig. 1), the cut-off values that produced ideal sensitivity and specificity were ≤ / > 59 steps during the 6MST (sensitivity = 72% and specificity = 52%). When we evaluated the applicability of this threshold over a 36-month period using the Kaplan Meier analysis (Fig. 2), significant event differences between subgroups was identified (log-rank test, Breslow and Tarone-Ware p < 0.05).

**Table 3:** Cut-off value, sensitivity and specificity of the number of steps in 6MST for exacerbation in COPD patients.

Variable	Cutt-off	COPD (n = 64)					
		Sensitivity	Specificity	AUC [CI–95%]	Youden index	Positive likelihood	Negative likelihood
Steps – 6MST	< 59	0.72	0.52	0.718 [0.586–0.849]	0.25	1.50	0.54

6MST: six-minute step test; COPD: chronic obstructive pulmonary disease; AUC: area under curve; CI: confidence interval.

The Cox regression model (Table 4) revealed that 6MST steps (CI 95% 0.934**–**0.987), gender (CI 95% 1.368**–**31.873), body fat mass (kg) (CI 95% 0.797**–**0.977) and total body mass (kg) (CI 95% 1.025**–**1.185) were all predictors of exacerbation risk.

**Table 4:** Risk factor for exacerbation in COPD (n = 64).

Covariates	Coefficient	Standard error	Hazard ratio (CI 95%)	*p* value
Number of steps by 6MST	− 0.040	0.014	0.960 (0.934–0.987)	< 0.01
Gender (male = 1; female = 0)	1.888	0.803	6.603 (1.368–31.873)	0.02
Total body mass (kg)	0.097	0.037	1.102 (1.124–1.185)	< 0.01
Body fat mass(kg)	− 0.125	0.052	0.882 (0.797–0.977)	0.02

6MST: six-minute step test; kg: kilos; meter; CI: confidence interval.

### Ethical approval and consent to participate

Approval by the Research Ethics Committee of the Federal University of São Carlos (UFSCar), protocol number: 5.188.654. All participants who voluntarily agreed to participate signed a consent form.

## Discussion

The originality of this investigation is linked to some important novel aspects: (1) a minimum number of steps reflected on the 6MST was identified as a strong predictor of exacerbations in individuals with COPD; (2) gender, body fat mass (kg) and total body mass (kg) can influence the prediction of exacerbations; and (3) a number of measures of clinical status significantly differed according to this 6MST threshold.

The use of a test involving going up and down a step as a clinical tool for evaluating outcomes was first proposed almost one century ago. Master et al.^22^ were pioneers in developing and use of a protocol that consisted of going up and down a platform with two steps, for a pre-established time of ninety seconds, so that the induction of coronary ischemia could be investigated through an electrocardiogram during exercise. Since then, the test has undergone several modifications and applications to different scenarios with a wide variety of uses, including the assessment of functional capacity^11,23^, fatigue^14^, dyspnea^14^ and exercise oxygen desaturation^24^. Operationally, one of the great advantages of the 6MST is its methodological simplicity. Considering its simplicity combined with its ability to reflect functional capacity, , the 6MST has been gaining popularity as an exercise assessment option in in different populations^11,13,23–25^ when advanced clinical exercise testing and space for the 6MWT are not feasible.

Discriminatingly, reduced functional capacity in individuals with COPD is common when compared to healthy individuals and reflects disease severity as well as physical inactivity^26^. Contrary to what may be assumed, this reduction in the level of physical activity and consequent reduction in functional capacity is not a characteristic linked solely to the moderate or advanced stage of the pathology, since evidence has shown that reduced physical inactivity can be observed at lower levels of disease severity and may even precede the onset of disabling symptoms^26^. As if that were not enough, in addition to implying a reduction in functional capacity, physical inactivity has been considered a strong predictor of unfavorable outcomes in COPD, such as exacerbation and hospitalization^27,28^, due to factors such as a progressive decline in lung function^29^, systemic inflammation^30^ and generalized muscle weakness^31^, in addition to the risk of all-cause mortality^29^. The results of the present study indicate a 6MST threshold of ≤ 59 steps may be clinically significant in identifying poorer overall functional/clinical status and prognosis.

In this way, identifying individuals prone to exacerbation, tracking and directing effective therapies to avoid the deleterious effects triggered by the worsening of the pathological condition remains a challenge, and despite the substantial costs invested in the health system for the treatment of the disease^32^, the models of evaluation and follow-up need to be strengthened to avoid decompensations and hospitalizations^33^. To this point, our findings highlight the potential valuable contribution of the 6MST: in addition to supporting the identification of those with reduced functional capacity^11^, we see here that its use can possibly identify and predict the risk of exacerbation in this population. However, despite having relatively good sensitivity,, the 6MST seems to be not very specific, and this leads us to believe that its use in clinical practice with the purpose of screening individuals prone to exacerbation should be associated with the combination of a multidimensional evaluation and should be considered before any therapeutic decision. Still, it is it is quite possible that the heterogeneous spectrum of severity that COPD has can directly interfere with sensitivity and specificity values. Considering that the appearance of more disabling signs and symptoms capable of influencing the triggering of the exacerbation are linked to more severe levels of severity, it is intuitive to imagine that the number of true positives in a relatively more severe population, whose functional capacity may be more impaired, would produce a higher sensitivity estimate. Likewise, considering only the reported accuracy in the specificity of the test can lead to inaccuracy, thus reaffirming the need for a multidimensional assessment. In addition, the arsenal of evaluative results that the 6MST can provide is not limited to the previously mentioned results, as this tool can also be used in the evaluation of disabling signs and symptoms, such as dyspnea and fatigue^34^, or even assessing the physiologic responses to exercise^35^ that reflect on the dynamics of heart rate, blood pressure and peripheral oxygen saturation^24^.

The reduction in functional capacity expressed in our results can be observed in both groups when considering the cutoff point previously defined by Pessoa et al.^11^ and the number of steps estimated from the equations proposed by Arcuri et al.^13^ and Albuquerque et al.^14^. In addition to these aspects, the group that performed ≤ 59 steps had worse lung function, especially in FEV_1_ and, particularly, the literature has already consolidated the observation that the risk of exacerbation and mortality are inversely proportional to this variable. Interestingly, the group that performed worse in the 6MST had a significantly higher proportion of individuals with worse dyspnea sensation when evaluated by mMRC, higher severity when classified by GOLD, in addition to a greater number of individuals who exacerbated over the 36 months of follow-up.

The association between reduced exercise capacity and the risk of mortality and exacerbation has been previously demonstrated^7,36^. Marino et al.^36^ observed that individuals undergoing the 6MWT whose distance covered was less than 340 m had a 30% greater risk of exacerbation when compared to the group that covered a distance greater than 357 m and 95% of those who walked more than 500 m along of six months of follow-up. The results previously described, parallel to ours, show the innovative importance of using a simple test, of low cost and of easy methodology in the prediction of unfavorable outcomes in individuals with COPD. Like the 6MWT, the 6MST has increasingly demonstrated its discriminative importance of functional capacity, relying on methodological characteristics as simple as the 6MWT.

## Clinical implications

Clinically, considering that exacerbations negatively contribute to the reduction of functional capacity^37^ and that this, in turn, cooperates so that exacerbations are triggered^38^, having low-cost and easily accessible tools, such as the 6MST, allows clinicians and scientists involved in screening the health status of individuals with COPD to perform a clinically important outcome results using less space, in less time, at a significantly lower operational cost. Furthermore, this type of approach can contribute to an effective screening right in primary care and that individuals with lower exercise performance are identified and directed to specific therapies, in addition to the benefit of being used as an evaluative strategy for follow-up over time.

## Strengths and limitations

This is the first study in the literature that investigated the predictive value of 6MST, an easily applied, valid and reproducible tool, on exacerbation outcome in individuals with COPD. However, this study has some limitations that will serve as an incentive for future studies to be developed: (1) all severities were considered here in this study. Although individuals with worse severity are more prone to exacerbations, the literature has described factors that may influence exacerbations in individuals with lesser severity. (2) Results in a more severe population should be further explored; (3) the same dataset was used to identify the cut-off point and analyze the data; (4) as a predictive tool, the Youden’s J index may not be suited for the tasks it balances sensitivity and specificity. For screening purposes, clinicians may prefer higher sensitivity than specificity.

## Conclusion

A minimal number of steps obtained through the 6MST can predict the risk of exacerbations in patients with COPD over 36 months of follow-up. Furthermore, factors such as body mass, sex and body fat mass can influence the test performance and contribute to the prediction of exacerbations in this population.

## Data Availability

The datasets used and/or analyzed during the current study are available from the corresponding author on reasonable request.
